# Alterations in acid–base balance and high-intensity exercise performance after short-term and long-term exposure to acute normobaric hypoxic conditions

**DOI:** 10.1038/s41598-020-70762-z

**Published:** 2020-08-13

**Authors:** Mirjam Limmer, Markus de Marées, Petra Platen

**Affiliations:** 1 Institute of Sports Medicine and Sports Nutrition, Ruhr-University Bochum, Bochum, Germany; 2 Institute of Outdoor Sports and Environmental Science, German Sports University Cologne, Cologne, Germany

**Keywords:** Metabolism, Fatigue, Hypoxia, Kidney, Physiology

## Abstract

This investigation assessed the course of renal compensation of hypoxia-induced respiratory alkalosis by elimination of bicarbonate ions and impairments in anaerobic exercise after different durations of hypoxic exposure. Study A: 16 participants underwent a resting 12-h exposure to normobaric hypoxia (3,000 m). Blood gas analysis was assessed hourly. While blood pH was significantly increased, PO_2_, PCO_2_, and SaO_2_ were decreased within the first hour of hypoxia, and changes remained consistent. A substantial reduction in [HCO_3_^−^] levels was observed after 12 h of hypoxic exposure (− 1.35 ± 0.29 mmol/L, *p* ≤ 0.05). Study B: 24 participants performed in a randomized, cross-over trial portable tethered sprint running (PTSR) tests under normoxia and after either 1 h (*n* = 12) or 12 h (*n* = 12) of normobaric hypoxia (3,000 m). No differences occurred for PTSR-related performance parameters, but the reduction in blood lactate levels was greater after 12 h compared with 1 h (− 1.9 ± 2.2 vs 0.0 ± 2.3 mmol/L, *p* ≤ 0.05). These results indicate uncompensated respiratory alkalosis after 12 h of hypoxia and similar impairment of high-intensity exercise after 1 and 12 h of hypoxic exposure, despite a greater reduction in blood lactate responses after 12 h compared with 1 h of hypoxic exposure.

## Introduction

Hypoxia has a profound influence on acid–base balance whenever unacclimatized people ascend to high altitudes. Barometric pressure decreases with increasing altitude and, therefore, oxygen pressure in the ambient and inspired air (P_I_O_2_) falls^1^. A reduced P_I_O_2_ leads to a decrease in arterial oxygen partial pressure (PO_2_) and to hypoxemia, which stimulates the peripheral chemoreceptors to evoke carbon dioxide (CO_2_) washout^2–4^. Concurrently, hyperventilation occurs as an hypoxic ventilatory response during acclimatization to high altitude, CO_2_ partial pressure (PCO_2_) falls, and arterial pH increases according to the Henderson–Hasselbalch equation^2, 5, 6^. This respiratory alkalosis is subsequently compensated by increased renal elimination of bicarbonate ions (HCO_3_^−^), which results in a decrease in blood bicarbonate concentration [HCO_3_^−^] and arterial pH returns to normal^2, 3, 5^. Metabolic compensation of respiratory alkalosis occurs after 6 h of altitude exposure and is completed after 24 h at low to moderate altitude^2, 7^. This compensation is further suggested to remain incomplete after 24 h of exposure to high altitude, but is then completed after several days^1, 2, 4, 7^. In contrast, lowlanders show persistent alkalosis even after 9 weeks at 5,260 m^8^. Nonetheless, to the best of our knowledge, information on values of pH and [HCO_3_^−^] during the first 24 h of altitude exposure is still insufficient.

[HCO_3_^−^] is an essential blood buffer for metabolic acids. During maximal workloads with blood lactate levels up to 15 mmol/L, there is a corresponding decrease in plasma [HCO_3_^−^] levels^9^. Therefore, the resulting decline in [HCO_3_^−^] and blood buffer capacity in the course of adaption to altitude might significantly affect exercise performance at altitude, particularly above the lactate threshold^2, 10–13^. Previous studies have investigated the effects of acute normobaric hypoxia on anaerobic performance parameters in experimental designs^14^. However, investigations on this research topic have provided inconsistent results. Some investigations reported significantly impaired anaerobic exercise performance when participants were exposed to acute normobaric hypoxia^15–21^, but others described constant anaerobic performance output under hypoxic conditions^22–26^. A possible explanation for these inconsistencies in reported results may be that no consistent study protocols were applied regarding the duration of exposure to hypoxic conditions before exercise. Most investigations only had in common that pre-exercise exposure to hypoxia mainly ranged between only 15 min and 1 h^15–17, 19, 27^. However, the renal response in hypoxia-induced respiratory alkalosis is considered to be a slow-adapting mechanism showing a significant reduction in blood [HCO_3_^−^] levels after several hours or days^2, 7^.

Actually, most alpinists who travel to medium and high altitudes, as well as athletes who do live-high-train-high altitude training, or participate in competitions at altitude are exposed to hypobaric hypoxic conditions for longer terms, at least several hours or days^28^. In addition, several normobaric hypoxic training strategies implicate lon-term or intermittent hypoxic exposure^14, 29, 30^. Therefore, the experimental set-up of short-term acute normobaric hypoxia applied in the above-mentioned studies might not have sufficiently considered the time course of renal compensation of hypoxia-induced respiratory alkalosis. Indeed, it has been reported that exercise performance partially or completely dependent on anaerobic energy metabolism is negatively affected by long-term altitude exposure^28, 31–36^. For instance, the performance of team sports in competitions at moderate to high altitude is impaired for teams living and training at sea level^28, 31, 32, 35^. In particular, high-intensity running and repeated sprint ability are negatively affected in soccer and rugby players competing at altitudes between 1,200 and 3,600 m above sea level^28, 31, 33^. Additionally, middle-distance runs (> 800 m) are dramatically impaired (2–4%) at heights of ≥ 1,000 m above sea level^34^. Hypoxia is also suggested to be a predictor for cross-country ski sprint performance^36^. These results indicate that anaerobic performance during sprint and team play trainings and competitions might be impaired because of hypoxic conditions at altitude. Moreover, performance in mountain sports disciplines performed at moderate to high altitudes and dependent on anaerobic exercise metabolism, such as alpine skiing, ski mountaineering, multi-pitch rock, mixed or ice climbing and mountain biking, might be negatively affected by hypoxic conditions^37^. Impaired anaerobic exercise performance in both athletes and mountaineers might be to a large extent a result of reduced blood [HCO_3_^−^] levels and an associated decline in blood buffer capacity. However, to the best of our knowledge, there is a lack of studies on the effect of different durations of hypoxic exposure on acid–base balance and anaerobic exercise performance.

Therefore, the present study aimed to investigate the effect of normobaric hypoxic conditions on blood gas analysis within the first 12 h during exposure to a simulated altitude. We further aimed to investigate whether there is a difference in anaerobic exercise performance, maximum capillary blood lactate concentrations, blood gas analysis parameters, and heart rate after either short-term (1 h) or long-term (12 h) exposure to acute normobaric hypoxic conditions. We hypothesized that extracellular buffering capacity represented by [HCO_3_^−^] gradually becomes reduced within 12 h of hypoxic exposure. Therefore, long-term exposure to hypoxia will affect anaerobic, high-intensity exercise performance, as well as associated physiological parameters, more than short-term hypoxic exposure.

## Methods

### Participants

All participants lived close to sea level and underwent medical screening before entering the study. Participants had to be moderately trained and in good health with no cardiac or pulmonary conditions. Additionally, with regard to Study B*,* participants had to be familiar with sprinting activities and have no musculoskeletal injuries that could interfere with running activities. Additional criteria for inclusion were as follows: (1) no preceding visits to altitude above 2,000 m within 4 weeks before the investigation, (2) a moderately active lifestyle (assessed by questionnaire), (3) no history of mental or physical impairment, (4) no history of smoking, and (5) no acute infections. The study protocols were conducted in accordance with the Declaration of Helsinki. The study was approved by the ethical committee of the Ruhr-University Bochum and by the ethics committee of the German Sports University Cologne. The participants were informed about experimental procedures, and the potential risks and benefits of the procedures involved, and provided written consent before starting the study.

*Study A* Sixteen participants aged 20–32 years voluntarily participated in this study. The means and standard deviations (SDs) for age, height, and body mass were 25.9 ± 3.0 years, 179.0 ± 3.3 cm, and 75.6 ± 3.4 kg, respectively, for men (*n* = 9), and 23.7 ± 3.6 years, 165.6 ± 4.6 cm, and 57.7 ± 3.5 kg, respectively, for women (*n* = 7). Two female Study A participants reported oral contraceptive ingestion (OCP).

*Study B* Twenty-four healthy, non-specifically trained adult volunteers (men: *n* = 12, women: *n* = 12) participated in this part of the study. Study B participants were randomly assigned to either the 1-h hypoxia group (G1, *n* = 12) or the 12-h hypoxia group (G12, *n* = 12) to assure high commitment to the study by reducing test days. The mean (± SD) age for G1 participants in Study B was 24.3 ± 2.0 years, with a mean height of 182.7 ± 4.4 cm and mean body mass of 83.3 ± 6.1 kg for male participants (*n* = 6). Mean age, height, and body mass were 24.3 ± 1.4 years, 171.5 ± 5.4 cm, and 63.4 ± 8.2 kg, respectively, for female participants (*n* = 6). For G12 participants in Study B, the mean (± SD) age was 25.5 ± 4.9 years, with a mean height of 180.2 ± 4.4 cm and mean body mass of 71.8 ± 6.1 kg for male participants (*n* = 6). Mean age, height, and body mass were 24.8 ± 3.5 years, 165.8 ± 4.9 cm, and 57.9 ± 5.6 kg, respectively, for female participants (*n* = 6).

### Experimental design

*Study A* All participants were exposed to a simulated altitude of 3,000 m for 12 h in a single experimental session. Study A participants were asked to perform only quiet and sedentary activities without any further activity specifications during the 12-h stay in a hypoxic chamber. Study A participants mainly preferred reading books, watching movies, working on laptops, playing board games, or conducting simple conversations. Blood gas analysis parameters, heart rate, food and fluid intake, amount of expelled urine, and urinary pH were assessed directly before entering the hypoxic chamber and every hour during the 12-h hypoxic exposure.

*Study B* The experimental protocol was completed in three visits separated by 48–72 h. All tests were performed indoors in a laboratory setting of a hypoxic chamber. Each visit occurred at approximately the same time of day to minimize the effects of diurnal variations on the measured variables. Study B participants were asked to avoid alcohol and caffeine ingestion, as well as additional sessions of heavy training during the experimental period that could interfere with the execution of sprinting. Additionally, Study B participants were asked to maintain their normal dietary habits and habitual lifestyle before and during the experimental period and not to exercise the day before the test trials to reduce interference from uncontrolled variables. On the first visit, each Study B participant was advised of the purpose, benefits, and risks associated with the study and underwent a familiarization trial for the test procedures under normoxic conditions. On the following two visits, Study B participants completed anaerobic performance tests under either normoxic (NOR) or hypoxic conditions (HYP) in a cross-over design. The participants were blinded to the hypoxic or normoxic condition. G1 and G12 participants in Study B performed the anaerobic performance tests in a hypoxic condition after exposition to a simulated altitude of 3,000 m for either 1 or 12 h. While G1 participants in Study B were asked to rest quietly for 1 h, G12 participants in Study B slept in the hypoxic chamber overnight. Anaerobic performance parameters, blood lactate levels, blood gas analysis parameters, and heart rate were assessed directly before entering the hypoxic chamber and pre- and post-anaerobic performance testing.

### Hypoxic chamber

For hypoxic conditions, Study A and Study B participants were exposed to a simulated altitude of 3,000 m. Altitude was simulated through nitrogen injection using a nitrogen generator (VPSA S325 V16; van Amerongen, Tiel, The Netherlands) in a 65-m^3^ normobaric hypoxic chamber, which was located near sea level. To simulate an altitude of 3,000 m, the fraction of inspired oxygen (F_I_O_2_) was reduced to 15.0% O_2_ and O_2_ levels were observed continuously using a single gas detector (GasAlert Extreme, BW Technologies, Calgary, Canada). The room temperature in the hypoxic chamber was kept at a constant level of 21–23 °C using air conditioning (42 WKR 61; Carrier, Neuss, Germany).

### Blood gas analysis

Capillary blood samples (100 µL) were collected from a hyperemized earlobe and were analyzed using a blood gas analyzer (ABL80 FLEX CO-OX; Radiometer, Willich, Germany). The parameters of [HCO_3_^−^], base excess (BE), PO_2_, PCO_2_, arterial oxygen saturation (SaO_2_), and blood pH (pH_b_) were determined.

*Study A* Before each blood gas analysis was performed, the participants sat on a chair for 5 min. Blood gas analyses were conducted in normoxia before Study A participants entered the hypoxic chamber (baseline) and hourly under hypoxic conditions (HYP1–HYP12).

*Study B* Blood gas analyses were carried out before entering the hypoxic chamber (PRE HYP), and pre- and immediately post-portable tethered sprint running (PTSR) tests. Post-PTSR analyses were carried out within the first minute after performing the PTSR test.

### Urine parameters

*Study A* The amount of expelled urine (∑ urine) and associated urinary pH values were determined within 12 h of hypoxic exposure for each Study A participant. To measure ∑ urine, participants were asked to urinate in containers with a measuring scale. Values of urinary pH were measured using Neutralit pH-indicator strips (pH of 5.0–10.0) (Merck, Darmstadt, Germany) in each sample. The hypoxic chamber was not equipped with sanitary facilities. Therefore, a hypoxic generator displaying oxygen-reduced air was provided. When going to the toilet, Study A participants wore a silicon mask that was connected to an oxygen-depleting respiratory system (b-cat High Altitude Generator 6,000; Tiel, The Netherlands) and breathed through a low-resistance two-way respiratory valve. The F_I_O_2_ also consisted of 15.0% oxygen, which is equivalent to an altitude of 3,000 m in the hypoxic chamber.

### Heart rate

*Study A* Heart rate was determined hourly. Study A participants sat on a chair for 5 min before heart rate was assessed using a finger-clip pulse oximeter (OXY500FB; Trismed, Daejeon, Korea) just before blood gas analysis was performed.

*Study B* Heart rate was recorded as a control parameter throughout the tests using a heart rate monitor (Polar T31; Polar Electro, Kempele, Finland). Therefore, heart rate was measured before exercise at rest, after warm-up, and after performance tests. Maximal post-exercise HR after performance tests was used for further analyses.

### Nutrition/food intake

*Study A* Participants had ad libitum access to foods and beverages during the experimental trial. To inhibit the metabolic effect of the acid–base status, foods were selected by a neutral classification according to their potential renal acid load (PRAL) as previously described^38^. The PRAL value is described in mEq/100 g and mainly ranged from ± 20 mEq/100 g. Foods and beverages within the range of ± 2.0 mEq/100 g were chosen in this study. Provided beverages were tea (− 0.3 mEq/100 g), coffee (− 1.4 mEq/100 g), and table water (− 0.1 mEq/100 g). Provided foods were wheat bread (1.8 mEq/100 g), butter (0.6 mEq/100 g), cream cheese (0.9 mEq/100 g), honey (− 0.3 mEq/100 g), jam (− 1.5 mEq/100 g), cucumber (− 0.8 mEq/100 g), peppers (− 1.4 mEq/100 g), dark chocolate (0.4 mEq/100 g), ice cream (0.6 mEq/100 g), fruit yoghurt (1.2 mEq/100 g), buttermilk (0.5 mEq/100 g), watermelon (− 1.9 mEq/100 g), and sugar (0.0 mEq/100 g). Additionally, after 5 h of altitude exposure, a warm meal consisting of refined white rice (1.7 mEq/100 g) and a cream sauce (1.2 mEq/100 g) containing mushrooms (− 1.4 mEq/100 g), leeks (− 1.8 mEq/100 g), peppers (− 1.4 mEq/100 g), and tofu (− 0.8 mEq/100 g) was offered to Study A participants. The food and fluid intake was controlled within the 12 h of the experimental trial by protocol. Overall fluid intake (∑ fluid), caloric intake (∑ CAL), and the PRAL value were calculated for each Study A participant.

### Anaerobic performance test

*Study B* Anaerobic performance was assessed using the PTSR test^39^. For this test, Study B participants ran with a belt round their waist for force measurements. The belt was attached to an inextensible static rope combined in series with a load cell and fixed to a pillar at a 90° angle to the participant’s waist height. Standardized and structured warm-ups were performed for 10 min before each test, including 5 min of light jogging on a treadmill, and 5 min of coordination and light dynamic stretching. Following the warm-up, “ready”, “set”, and “go” commands were provided and the participant performed an all-out sprint for 60 s. Each Study B participant was instructed to perform the sprinting maximally and to pull the rope with full force until voluntary exhaustion. Study investigators provided strong verbal encouragement for the duration of the entire test. Force data were recorded in newtons (N) and downloaded to an online PC using a sampling rate of 100 Hz. Overall peak force (PF), overall mean force (MF), and the fatigue index (FI) over 60 s were determined for subsequent analysis. The FI was calculated by following the recommended calculations for Wingate tests as follows: FI (%) = [(PF − F_min_)/PF]/100^40^. For measurement of blood lactate levels, 20-µL capillary blood samples were collected before and 2, 4, 6, 8, and 10 min after PTSR testing. Blood lactate measurements were conducted directly after collection of blood samples (Biosen S-Line; EKF-diagnostic, Magdeburg, Germany) and the maximum post-exercise lactate concentration (La_max_) was used for statistical analyses.

### Anthropometric characteristics

Body weight was determined with a sliding weight mechanical scale (Seca 709, Seca, Hamburg, Germany). Height was measured (to the nearest 0.1 cm) using a scale-integrated stadiometer.

### Statistical analysis

Data are presented as mean ± SD. A non-normal distribution was identified using the Shapiro–Wilk test. The significance level was set a priori at *p* ≤ 0.05 for all comparisons. Statistical analyses were performed using the statistical data analysis program SPSS 25 (IBM Corp., Armonk, NY, USA). The free software G*Power (version 3.1.9.4; https://www.gpower.hhu.de/) was used to calculate the required sample sizes and effect sizes^41^.

*Study A* The differences in [HCO_3_^−^], BE, PO_2_, PCO_2_, SaO_2_, pH_b_, and heart rate over time (NOR vs. HYP1–HYP12) were determined by one-way repeated measures ANOVA. Greenhouse–Geisser adjustments were used for correction if violation of the assumption of sphericity occurred. Two-tailed *t *tests were used as post hoc tests to indicate significant differences and the Bonferroni procedure was used (p*) to retain α = 0.05. Effect sizes were calculated using partial η squared (ηp^2^) and interpreted as small (0.01), medium (0.06), or large (0.14). To further determine which variables might be predictors for a reduction in [HCO_3_^−^] under hypoxic conditions (Δ [HCO_3_^−^] = [HCO_3_^−^] HYP 12 − [HCO_3_^−^] baseline), we performed stepwise multiple linear regression analyses. We entered nutritional variables (∑ CAL, ∑ fluid, and PRAL), ∑ urine, baseline pH_b_, and sex of participants into the model. Additionally, we computed the acute change in heart rate, SaO_2_, and PO_2_ after entering the hypoxic chamber (Δ = HYP1 – baseline) and entered it into stepwise multiple linear regression analysis. Differences in Δ [HCO_3_^−^] values between male and female Study A participants were assessed using the two-sample *t *test. An a priori* analysis* indicated a required sample size of *n* = 14 to detect significant differences in [HCO_3_^−^] values based on an estimated α level of 0.05 with a power of at least 0.8 (based on within-subject changes in [HCO_3_^−^] from 23.5 ± 2.5 mmol/L in normoxic conditions to 21.7 ± 2.5 mmol/L at an altitude of 3,000 m from a previous study)^5^.

*Study B* We calculated ∆ values (HYP − NOR) for all PTSR-related parameters to identify the effect of hypoxia on these parameters. Differences in ∆ values between G1 and G12 and between male and female Study B participants were assessed using the two-sample *t *test. Cohen’s d (*d*) was used to calculate effect sizes, with 0.2 considered to indicate a small effect, 0.5 a medium effect, and 0.8 a large effect^42^. Non-normally distributed variables (∆ PF, ∆ MF, ∆ heart rate) were analyzed using the Mann–Whitney-U-test and effect sizes were calculated using correlation coefficients (*r*). Paired sample *t *tests were calculated for pairwise comparisons of PTSR-related parameters under the conditions of normoxia and hypoxia for G1 and G12 and between male and female Study B participants. Wilcoxon tests were used when variables were not normally distributed (MF under NOR). The effects of conditions (G1 and G12; male and female) and time (pre- and post-PTSR test under NOR; PRE HYP, and pre- and post-PTSR test under HYP) on the parameters [HCO_3_^–^], BE, PO_2_, PCO_2_, SaO_2_, and pH_b_ were tested by two-way (condition × time) repeated-measures ANOVA. Violations of the assumption of sphericity were corrected for by Greenhouse–Geisser adjustments. The two-tailed *t *test was used as a post hoc test to indicate significant differences. The Bonferroni procedure was used (p*) to retain α = 0.05 and the significance level was set at *p* ≤ 0.05 in all comparisons. Effect sizes were calculated using ηp^2^. An a priori power calculation indicated that six participants per group were required to detect significant differences in performance outputs based on an estimated α level of 0.05 and a power of 90% (based on anaerobic performance reduction results after exposure to a simulated altitude of 3,000 m from a previous study)^21^.

## Results

### Study A

#### Blood gas analysis

**Figure 1:**
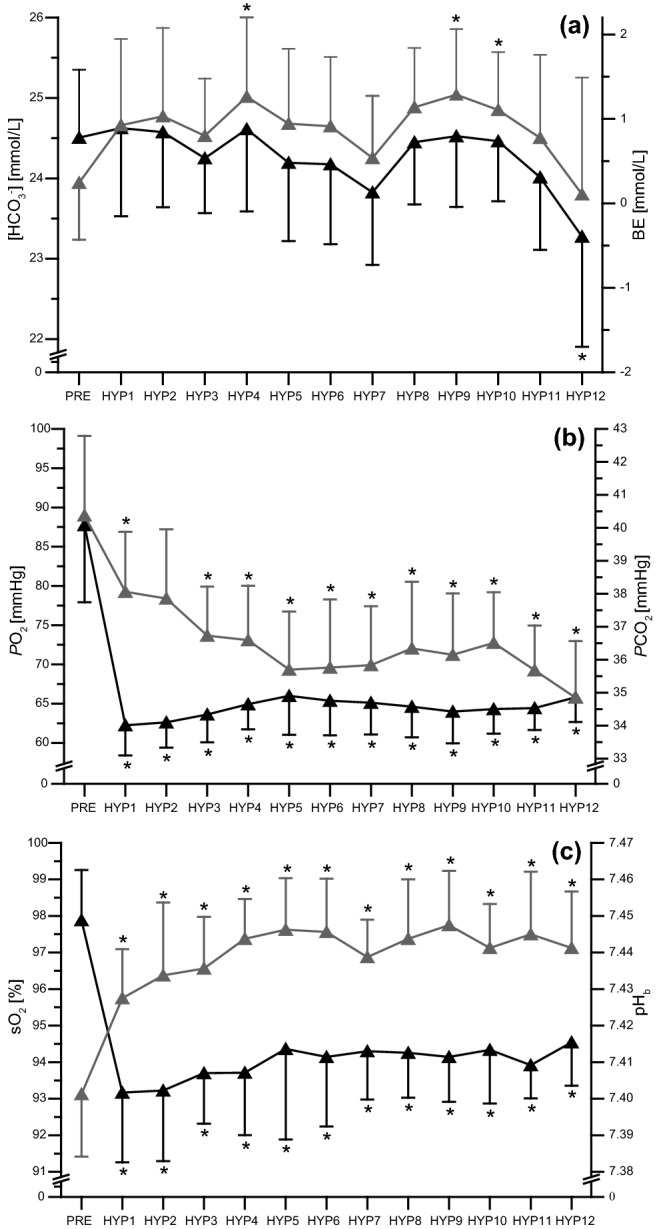
Study A: time course for (**a**) blood bicarbonate concentration ([HCO_3_^**−**^]) and active base excess (BE), (**b**) oxygen partial pressure (PO_2_) and carbon dioxide partial pressure (PCO_2_), and (**c**) arterial oxygen saturation (SaO_2_) and blood pH (pH_b_) under normoxic conditions (PRE) and during 12 h of exposure to a simulated altitude of 3,000 m (HYP1–HYP12) in participants of Study A (*n* = 16). Data points represent mean ± standard deviation for [HCO_3_^**−**^], PO_2_, and SaO_2_ (filled triangle), and BE, PCO_2_, and pH_b_ (filled riangle). See “Methods” section for further details. *p ≤ 0.05 compared with PRE.

A significant time effect was observed for [HCO_3_^−^] (*p* < 0.001, ηp^2^ = 0.306). Post hoc analysis showed a significant decrease in [HCO_3_^−^] from HYP12 to baseline. We also observed a significant time effect for BE (*p* < 0.001, ηp^2^ = 0.253) and post hoc analysis showed an increase between the baseline value of BE and those of HYP5, 10, and 11 (Fig. 1a). Further, PO_2_ (*p* < 0.001, ηp^2^ = 0.759), PCO_2_ (*p* < 0.001, ηp^2^ = 0.534), and SaO_2_ (*p* < 0.001, ηp^2^ = 0.437) were significantly decreased under hypoxic conditions and remained reduced during the whole experimental period (Fig. 1b, c). In contrast, pH_b_ (*p* < 0.001, ηp^2^ = 0.470) was significantly increased under hypoxic conditions and remained elevated for up to 12 h of hypoxic exposure (Fig. 1c).

#### Heart rate

For heart rate, we observed a significant time effect (*p* = 0.003, ηp^2^ = 0.150). Post hoc analysis showed a significant increase in heart rate from baseline (66.1 ± 10.5 bpm) after 4 h (75.1 ± 10.0 bpm; *p* = 0.005, *d* = 0.90) and 7 h (75.8 ± 10.2 bpm; *p* = 0.011, *d* = 0.97) of hypoxic exposure.

#### Multiple regression analyses

The results of multiple linear regression analysis on a reduction in [HCO_3_^−^] under hypoxic conditions are shown in Table 1. Sex was identified as a significant predictor for a reduction in [HCO_3_^−^] under hypoxic conditions. However, the variables ∑ CAL (2,360.5 ± 623.1 kcal), PRAL (− 1.2 ± 4.0 mEq), ∑ fluid (3,012.5 ± 843.5 mL), ∑ urine (3,240.3 ± 1,070.4 mL), baseline pH_b_ (7.40 ± 0.02), Δ SaO_2_ (− 4.7% ± 1.6%), Δ PO_2_ (− 25.5 ± 7.6 mmHg), and ΔHR (4.4 ± 10.2 bpm) did not significantly predict a reduction in [HCO_3_^−^] under hypoxic conditions (Table 1).

**Table 1:** Linear multiple regression analysis on the reduction in blood bicarbonate concentrations.

Predictor variable	R^2^	Corrected R^2^	F	*p*	Standardized β	*t*	*p*
Model	0.346	0.299	7.407	0.017*			
Sex					0.588	2.722	0.017*
∑ CAL					0.256	1.071	0.304
PRAL					0.072	0.321	0.754
∑ fluid					0.404	2.023	0.064
∑ urine					0.333	1.629	0.127
pH_b_ baseline					0.139	0.527	0.607
ΔSaO_2_					0.302	1.439	0.174
ΔPO_2_					0.216	0.986	0.342
ΔHR					− 0.105	− 0.459	0.654

The linear multiple regression model of the reduction in blood bicarbonate concentration ([HCO_3_^−^]) (Δ [HCO_3_^−^] = [HCO_3_^−^] HYP12 − [HCO_3_^−^] baseline) included the sex of participants, overall caloric intake (∑ CAL), associated potential renal acid load (PRAL), overall fluid intake (∑ fluid), amount of expelled urine (∑ urine), baseline blood pH (pH_b_) values, the acute change in oxygen saturation (SaO_2_) (ΔSaO_2_ = SaO_2_ HYP1 − SaO_2_ baseline), the acute change in oxygen partial pressure (PO_2_) (ΔPO_2_ = PO_2_ HYP1 − PO_2_ baseline), and the acute change in heart rate (ΔHR = HR HYP1 − HR baseline) (*n* = 16). *p ≤ 0.05.

**Figure 2:**
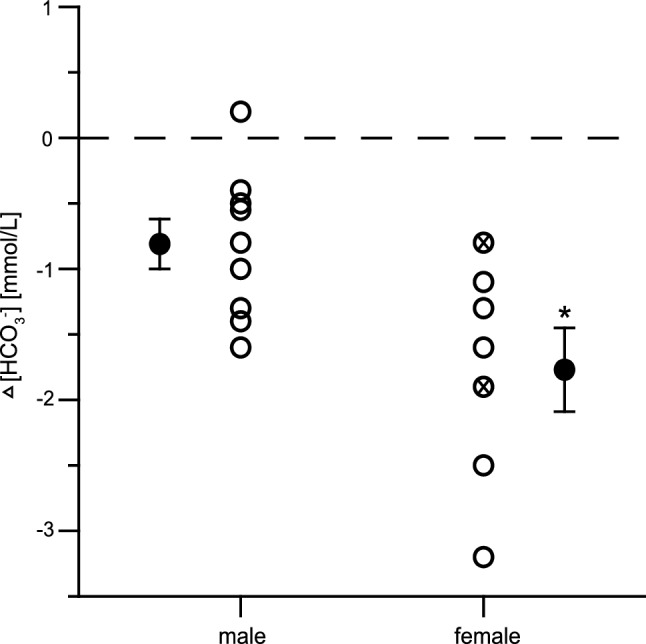
Study A: reduction in blood bicarbonate concentration ([HCO_3_^**−**^]) after 12 h of exposure to a simulated altitude of 3,000 m (Δ [HCO_3_^**−**^] = [HCO_3_^**−**^] HYP12 − [HCO_3_^**−**^] PRE) in male (*n* = 9) and female (*n* = 7) participants. Data points represent individual values (open circle) and mean ± SD (filled circle) for Δ [HCO_3_^**−**^]. An “x” indicates female participants with oral contraceptive ingestion. See “Methods” section for further details. *p ≤ 0.05 compared with the corresponding values in male participants.

Comparison of Δ [HCO_3_^−^] between male and female Study A participants showed a more pronounced reduction in [HCO_3_^−^] for female participants than male participants (*p* = 0.017, *d* = 2.52) (Fig. 2).

### Study B

#### Anaerobic performance test

**Figure 3:**
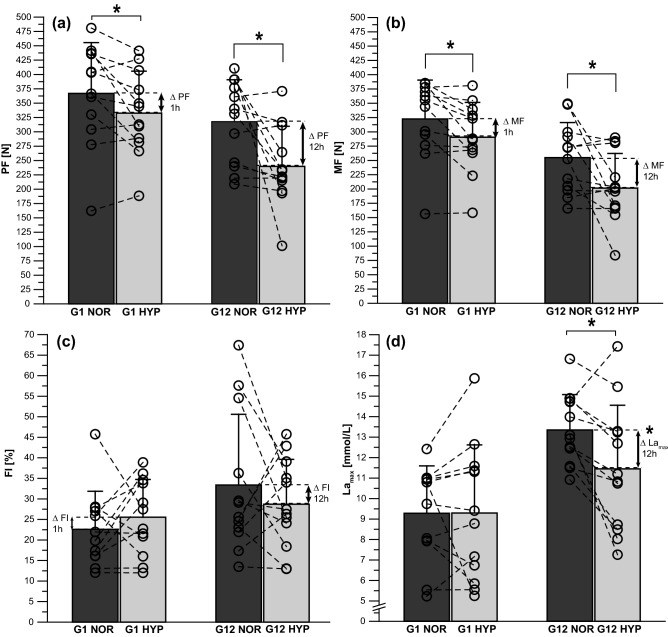
Study B: performance measurements in the 1-h hypoxia group (G1) (*n* = 12) and the 12-h hypoxia group (G12) (*n* = 12) under normoxia (NOR) and after 1 or 12 h of hypoxic exposure (HYP), respectively, for (**a**) peak force (PF), (**b**) mean force (MF), (**c**) the fatigue index (FI), and the associated physiological response of (**d**) maximum post-exercise lactate concentration (La_max_). ∆ values (HYP − NOR) indicate intra-individual hypoxic-induced changes in performance parameters. Data points represent individual values (open circle). Bar charts show mean ± standard deviation. *p ≤ 0.05 compared with NOR. See “Methods” section for further details.

Hypoxia had a detrimental effect on the PTSR-related performance output of PF, MF, and La_max_ (all *p* < 0.05) (Fig. 3a, b, d). However, there were no significant differences in hypoxia-induced performance changes in ∆PF (G1: − 34.5 ± 49.6 N, G12: − 77.9 ± 99.8 N; *p* = 0.410, *r* = 0.38), ∆MF (G1: − 32.0 ± 36.2 N, G12: − 53.4 ± 74.6 N; *p* = 0.887, *r* = 0.30), ∆FI (G1: 2.9% ± 11.2%, G12: − 4.7% ± 17.6%; *p* = 0.220, *d* = 0.52) (Fig. 3a, b, c), and ∆HR (G1: − 1.3 ± 4.7 bpm, G12: 4.3 ± 18.1 bpm; *p* = 0.932, *r* = 0.02) between G1 and G12 participants in Study B. Significant differences were only found in ∆La_max_ (G1: 0.0 ± 2.3 mmol/L, G12: − 1.9 ± 2.2 mmol/L; *p* = 0.045, *d* = 0.84) between the groups (Fig. 3d). There were no significant differences in HR or any of the PTSR-related parameters between male and female Study B participants.

#### Blood gas analysis

Significant main effects (time) were found for all blood gas analysis parameters, which indicated that hypoxia had an overall influence on these parameters. A significant condition × time interaction was detected in G1 and G12 for PCO_2_ (*p* = 0.025, ηp^2^ = 0.142), PO_2_ (*p* < 0.001, ηp^2^ = 0.296), SaO_2_ (*p* = 0.008, ηp^2^ = 0.226), pH_b_ (*p* = 0.001, ηp^2^ = 0.180), and BE (*p* = 0.044, ηp^2^ = 0.129), but not for [HCO_3_^−^] (*p* = 0.069, ηp^2^ = 0.110). Post hoc analyses results are shown in Table 2. There were no significant differences in any of the blood gas parameters between male and female participants.

**Table 2:** PTSR-related blood gas parameters in G1 and G12 in Study B under normoxia and after 1 or 12 h of hypoxic exposure, respectively.

	PO_2_ (mmHg)	PCO_2_ (mmHg)	SaO_2_ (%)	pHb	[HCO_3_−] (mmol/L)	BE (mmol/L)
**NOR**						
*G1*						
PRE PTSR	85.7 ± 8.1^#^	41.2 ± 2.6	97.0 ± 0.8^#^	7.40 ± 0.02	24.7 ± 2.0	0.3 ± 1.9
POST PTSR	88.3 ± 4.9^#^	41.6 ± 3.1	96.0 ± 0.8^#^	7.26 ± 0.05	18.2 ± 2.3	− 0.2 ± 1.9
*G12*						
PRE PTSR	84.7 ± 6.1^#^	37.8 ± 2.6^*^	98.3 ± 0.8^*#^	7.41 ± 0.02	23.6 ± 1.3	− 0.2 ± 1.1
POST PTSR	92.9 ± 10.0^#^	42.2 ± 5.5	97.2 ± 1.1^*#^	7.21 ± 0.05	16.3 ± 2.4	− 10.7 ± 2.9
**HYP**						
*G1*						
PRE HYP	80.1 ± 8.0	41.1 ± 2.5	96.0 ± 1.3	7.40 ± 0.02	24.8 ± 1.4	0.3 ± 1.9
PRE PTSR	54.6 ± 3.5	39.5 ± 2.5	89.3 ± 1.4	7.41 ± 0.02	24.2 ± 1.7	0.1 ± 1.6
POST PTSR	53.5 ± 4.2	38.9 ± 3.6	85.7 ± 4.9	7.28 ± 0.08	17.8 ± 2.5	− 8.1 ± 3.9
*G12*						
PRE HYP	90.8 ± 7.8^*^	40.5 ± 3.1	98.6 ± 0.9^*^	7.41 ± 0.01	24.8 ± 1.8	0.6 ± 1.4
PRE PTSR	67.3 ± 3.7^*^	36.7 ± 3.8	93.6 ± 1.2^*^	7.42 ± 0.02	23.5 ± 1.7	− 0.4 ± 1.5
POST PTSR	70.3 ± 3.8^*^	40.0 ± 5.8	91.1 ± 2.4^*^	7.24 ± 0.06	16.5 ± 1.8	− 10.3 ± 2.6

Data are shown as mean ± standard deviation. PO_2_ = oxygen partial pressure; PCO_2_ = carbon dioxide partial pressure; SaO_2_ = oxygen saturation; pH_b_ = blood pH; [HCO_3_^–^] = blood bicarbonate concentration; BE = base excess; G1 = 1-h hypoxia group (*n* = 12); G12 = 12-h hypoxia group (*n* = 12); NOR = normoxia, HYP = hypoxia, PTSR = portable tethered sprint running test; PRE PTSR = pre-PTSR values; POST PTSR = post-PTSR values. For further details see the “Methods” section. ^*^p < 0.05 versus G1, ^#^*p* < 0.05 versus HYP.

## Discussion

The present study was composed of two separate investigations. In Study A*,* we investigated the hourly changes in acid–base status during acute exposure to a simulated altitude of 3,000 m and possible predictors for early HCO_3_^−^ loss resulting from a hypoxic ventilatory response. Our primary findings are as follows: (1) [HCO_3_^−^] was significantly reduced after 12 h of hypoxic exposure to a simulated altitude of 3,000 m; and (2) sex was a significant predictor of a reduction in [HCO_3_^−^] under hypoxic conditions where there was a greater reduction in [HCO_3_^−^] in female participants than in male participants. In Study B, the effect of 1 or 12 h of hypoxic exposure to a simulated altitude of 3,000 m on high-intensity exercise (represented by PTSR test performance outcomes) compared with sea level performance was investigated. Exposure to hypoxic conditions generally resulted in impairment of high-intensity exercise performance, regardless of the duration of hypoxic exposure. However, the effect of hypoxia on PTSR-related performance parameters, as represented by ∆ values, was not different between 1 and 12 h of hypoxic exposure. However, after 12 h of hypoxic exposure, a reduction in blood lactate levels was observed, whereas 1 h of hypoxia did not result in a change in blood lactate levels. Therefore, the effect of hypoxia on metabolic responses to high-intensity exercise differed depending on the duration of hypoxic exposure.

### Blood gas analysis and blood buffer capacity

Hypoxia leads to respiratory alkalosis, which is compensated for by renal elimination of HCO_3_^−^, and [HCO_3_^−^] is an essential blood buffer for metabolic acids^2, 3^. Uncompensated respiratory alkalosis is associated with a reduction in the diagnostic parameters PCO_2_ and [HCO_3_^−^] in combination with increased pH_b_ and constant BE. Further, hypoxia leads to a decrease in SaO_2_ and PO_2_^2^. In Study A, an expected hypoxia-induced decrease in PO_2_, PCO_2_, and SaO_2_, and an increase in pH_b_ were observed. Values of pH_b_ increased within the first hour of altitude exposure and remained elevated over 12 h of altitude exposure. During acclimatization to high altitudes, metabolic compensation for respiratory alkalosis occurs, resulting in a slow return of pH_b_ to its normal level. An example of this situation is that a completely compensated mean pH_b_ of 7.40 ± 0.01 was reported for high-altitude residents at an altitude of 3,500 m^4^. However, lowlanders usually have a slightly alkaline pH during acclimatization, even after 10 days of acclimatization^2^. Measured values within the present investigation (Study A) showed the expected increase in pH_b_ resulting from hypoxia-induced respiratory alkalosis. However, there was no trend towards a decrease in pH back to normal associated with a compensatory metabolic acidosis, as previously reported^1, 3^.

With regard to hypoxia-induced changes in [HCO_3_^−^] after several hours of altitude exposure, the kidney responds to a decline in PCO_2_ and an increase in pH_b_ through excretion of HCO_3_^−^ known as compensation of respiratory alkalosis. According to the literature, we expected a reduction in [HCO_3_^−^] by 1.8 ± 2.5 mmol/L and an [HCO_3_^−^] of 21.7 ± 2.5 mmol/L for acute 12-h exposure to an altitude of 3,000 m^5^, regardless of a normobaric or hypobaric acute hypoxic exposure^43, 44^. However, in Study A, after 12 h of hypoxic exposure, there was a significant reduction in [HCO_3_^−^] of only 1.4 ± 0.3 mmol/L and [HCO_3_^−^] was 23.3 ± 1.3 mmol/L. Therefore, the increased blood pH values and lower [HCO_3_^−^] in Study A only indicate uncompensated respiratory alkalosis. However, the present results on an ongoing, but insufficient, renal compensation of hypoxia-induced respiratory should be interpreted with caution. This finding is consistent with that reported by Ge et al.^7^ who analyzed urine acid–base compensation at simulated altitudes of 1,780, 2,085, 2,455, and 2,800 m. These authors showed that renal compensation had occurred after 6 h and was completed by 24 h up to an altitude of 2,455 m, but it remained insufficient at 2,800 m. However, the finding of an increase in BE during 12-h hypoxic exposure in Study A is in contrast to expected constant BE levels commonly associated with uncompensated respiratory alkalosis. We assume that consumed food and beverages, especially the meal that was served for lunch between the testing points of HYP6 and HYP7, could have contributed to metabolic changes in blood gas analysis parameters. The increase in BE and [HCO_3_^−^] following the ingested meal after testing point 7 may be attributed to the well-known *alkaline tide* phenomenon, a short-term post-prandial metabolic acidosis that occurs after eating a meal because of hydrochlorid acid production by parietal cells in the stomach and simultaneous basolateral HCO_3_^−^ excretion into the blood. In addition, food choices can lead to metabolic alterations in [HCO_3_^−^] and BE values due to their PRAL impact. However, we do not assume that provided foods, although potentially different from normal dietary habits of the participants, significantly affected blood gas analysis parameters within the present investigation (Study A) because they were selected by a neutral classification according to their PRAL value. Therefore, it might be speculated that there is a gradually reduction in BE and [HCO_3_^−^] in Study A participants which only was interrupted by the post-prandial increase in BE and [HCO_3_^−^] after testing point 7. However, this assumption has yet to be verified in future studies investigating hypoxia-induced acid–base changes over night or in a fasted state. Thus, the hypothesis of a gradually reduced extracellular buffering capacity as represented by [HCO_3_^−^] within 12 h of hypoxic exposure could not be confirmed in Study A. However, our finding of a reduction in [HCO_3_^−^] after 12 h of hypoxic exposure to a simulated altitude of 3,000 m suggests that high-intensity, anaerobic exercise performance might be reduced from this point because of reduced blood buffer capacity^2, 10–13^.

### Anaerobic, high-intensity exercise performance

Several studies have investigated the effects of acute hypoxia on anaerobic performance parameters^15–17, 19, 20, 23, 24^. To date, studies have provided inconsistent results regarding changes in anaerobic exercise performance with either a significantly impaired^15–20^ or constant^22–24, 45^ anaerobic exercise performance when exposed to acute hypoxia. These contrasting findings may result from methodological differences in applied study protocols, such as exercise type and intensity and the duration of exposure to hypoxic conditions before exercise^46^. The durations of hypoxic exposure before exercise testing mainly ranged between only 15 min and 1 h^15–17, 19, 27^. Therefore, the time course of the compensation process of hypoxia-induced respiratory alkalosis and possible effects of reduced [HCO_3_^−^] on high-intensity exercise were not focused on in these studies. Additionally, recent studies mainly used 30- and 45-s Wingate tests to assess anaerobic exercise performance^18, 20, 22–24, 45^. Furthermore, most of these studies did not show an impairment in power output following hypoxic exposure^22–24, 45^. Short duration, high-intensity exercise performance can be maintained in acute hypoxic conditions because of a shift toward anaerobic metabolism^46, 47^. In contrast, power output for tests with longer continuous or repeated high-intensity exercise, such as the 3-min all-out critical power test and repeated sprints, is reduced in acute hypoxia^15–17, 19, 27^. Proposed reasons for this impairment in performance are an increase in breathing frequency and perceived exertion, and limited muscle oxygen availability during recovery periods in these high-intensity sprint settings^15, 16, 46, 48^. Therefore, we decided to apply a 60-s continuous tethered sprint test to assess anaerobic, high-intensity exercise performance in Study B. We observed the expected impaired exercise performance as represented by MF and PF, although the hypothesized hypoxia-induced [HCO_3_^−^] loss did not result in differences in exercise performance parameters between short-term and long-term acute hypoxic exposure. Only blood lactate levels showed different responses with reduced maximal blood lactate levels after 12 h of hypoxic exposure compared with normoxic conditions. The blood lactate response to exercise at altitude is still controversial^10, 49^. However, an increased lactate response occurs for a given workload at acute exposure to altitude, whereas maximal lactate concentrations are reduced at the same relative workload during an acclimatization period^49^. The unexpectedly (paradoxically) low blood lactate concentrations during exercise at high altitude in acclimatized participants compared with participants exposed to acute hypoxia is called the lactate paradox^50^. This concept and multifactorial phenomenon has been intensively debated in the last decade^10, 50–53^. Our findings support the assumption of the lactate paradox that acclimatization processes lead to a reduction in maximum blood lactate levels. This is because while short-term acute exposure to hypoxia resulted in an increase in maximum blood lactate concentrations, long-term acute exposure of 12 h to hypoxia caused lower lactate responses in our study. Enhanced use of blood lactate by lactate-consuming organs, such as inactive muscles, the myocardium, or the kidneys, might be a reason for reduced lactate levels during the acclimatization process^10^. Cerretelli and Samaja^10^ concluded that downregulation of anaerobic glycolysis may contribute to decreased maximum lactate values for exercise testing up to voluntary exhaustion. Another mechanism explaining this phenomenon might be hypoxia-induced upregulation of red cell membrane proteins and several muscle-related proteins involved in the transport of bicarbonate, hydrogen ions, and lactate^54^. This leads to augmentation of the transport capacity of these ions and thus improves the dynamics of maintaining the acid–base balance at altitude^49^. The assumption that relatively lower maximum blood lactate values in long-term compared with short-term acute exposure may be attributed to ongoing acclimatization processes is supported by blood gas analysis results in Study B. An overnight stay in the hypoxic chamber led to an increase in PO_2_ and SaO_2_ values compared with short exposure to hypoxic conditions for only 1 h. This finding indicated physiological adaption to hypoxic stress.

Nevertheless, our study design and interpretation of results were within the classical Henderson–Hasselbalch approach, which presumes that blood pH is determined by changes in [H^+^] and [HCO_3_^−^]. However, the contrasting theory of strong ion difference (SID) may also have contributed to different blood gas analysis results and maximum blood lactate concentrations after short-term or long-term exposure to hypoxia in the present study^55^. The SID theory involves intracellular and extracellular ions (e.g., sodium, chloride, and potassium), and describes the difference between concentrations of strong cations and strong anions. An SID might also affect muscle performance by altering intracellular or extracellular pH because of an independent effect on blood pH^55^. A modified physicochemical approach has been suggested to offer more detailed insight into the complex changes in acid–base status during exercise in normoxia and hypoxia^56^. Therefore, the SID approach may also contribute to an explanation for the exercise-induced difference in La_max_ values after short-term and long-term hypoxic exposure with simultaneous similar changes in the PTSR-related parameters pH_b_, [HCO_3_^−^], and BE in the present study. However, this conclusion should be interpreted with caution because SID values were not calculated in our investigation. Future studies are required to address this limitation of the present study and to examine the effect of changes in an SID on high-intensity exercise performance under hypoxic conditions.

### Practical applications

Athletic competitions are contested at a wide range of terrestrial altitudes ranging from sea level to 3,600 m (La Paz, Bolivia)^15, 35^. The negative effect of moderate to high altitude on anaerobic exercise performance has been shown for soccer^31–33^, rugby^28^, cross-country ski sprinting^36^, and track and field^34^ in non-acclimatized athletes. For competitions at altitude, athletes travel to the competition destinations for several hours or days^28^. The present study showed a reduced blood buffer capacity beginning from 12 h after exposure to altitude and an impaired anaerobic exercise performance, regardless of the duration of acute hypoxic exposure. Normobaric hypoxia, which was used in the present investigation, is not as representative for real-life situations at high altitude as hypobaric hypoxia and transfer of the present results to hypobaric hypoxic situations should be taken with caution. However, athletes not only compete but also train at normobaric and hypobaric hypoxic conditions. In this context, the present results may be of special interest for normobaric hypoxic training strategies^14, 29, 30^. We suggest that coaches and athletes pay attention to this [HCO_3_^−^] loss with regard to possible associated performance impairments. Furthermore, future investigations about performance impairment related to the duration of altitude exposure might be interesting to support these assumptions. Additionally, blood gas analysis before competitions at altitude might be informative for coaches and athletes to detect causes of impairments of anaerobic exercise. Another practical approach, however not investigated within the present study, might be a pre-exercise sodium bicarbonate (NaHCO_3_) ingestion to delay exercise-induced acidosis and enhance the contribution of anaerobic energy. NaHCO_3_ is a well-known ergogenic aid that is used to improve high-intensity exercise in normoxia^57^. Pre-exercise NaHCO_3_ ingestion has a beneficial effect on intermittent and repeated high-intensity exercise under short-term normobaric hypoxia^27, 58^. However, further research is required to determine the usefulness of NaHCO_3_ on exercise.

### Sex as a predictor

Physiological responses to hypoxic conditions are similar between men and women, but there may be some differences in the magnitude of these responses^2^. The compensatory vasodilator response to exercise in hypoxia is assumed to be greater in women than in men^59^. Additionally, substantial acclimatization in pulmonary artery systolic pressure at rest has been shown to be 40% greater in women than in men after an 8-h exposure to hypoxia. Therefore, there might be a sex-specific difference in pulmonary vascular acclimatization, but not in ventilatory acclimatization^60^. However, PCO_2_ decreases more in women than in men during acclimatization to altitude^2, 61, 62^. These previous findings suggest that women have to compensate for a more severe respiratory alkalosis^62^. Our data in Study A support this assumption because we found greater [HCO_3_^−^] losses after 12 h at a simulated altitude of 3,000 m in female participants than in male participants. Therefore, our finding suggested a greater acid–base response to sustained hypoxia in women than in men. However, this greater blood [HCO_3_^−^] loss was not found in female Study B participants and did not result in greater impairment of exercise in women at altitude in our study. We attribute these deviating results in Study A and Study B for female participants to diurnal variations. Whereas Study A participants were assessed hourly during daytime, Study B participants slept in the hypoxic chamber overnight. Because minute ventilation and hypoxic ventilatory response are depressed during sleep compared to the awake state^2^, we attribute differences in results of Study A and Study B to an overall smaller respiratory and therewith acid–base response overnight. However, this assumption needs to be verified in future studies. Nevertheless, differences in PCO_2_ have frequently been observed between men and women. Additionally, an association between levels of progesterone and estrogen and differences in PCO_2_ have been found in the menstrual cycle and during pregnancy^62, 63^. Elevated progesterone levels in the luteal phase of the menstrual cycle phase and during pregnancy, or progestin in oral contraceptives, increases ventilation^62, 64^. Therefore, hormonal fluctuations might alter the ability to respond to hypoxia resulting from high altitude. However, progesterone–progestin variations are related to a higher ventilation in normoxia, but not at altitude^62^. In Study A, we evaluated OCP ingestion of our female participants. Two female participants with OCP ingestion showed no differences compared with women without OCP ingestion. However, we did not investigate differences in sex hormone levels in female Study A participants. Additionally, our sample was not sufficient in size to detect possible effects of OCP ingestion on acid–base disturbances in hypoxia. Therefore, the effect of these factors on changes in the acid–base status and impairment of anaerobic performance in women during acclimatization to high altitude remain to be established and might be of interest for future research.

## Conclusions

This is the first study to examine the effect of acute normobaric hypoxia on acid–base status at rest in young and healthy humans using hourly measurements for the first 12 h of hypoxic exposure. Our study shows a significant reduction in [HCO_3_^−^] and a maintained increase in pH_b_ after 12 h of hypoxic exposure to a simulated altitude of 3,000 m. This indicates an uncompensated respiratory alkalosis after 12 h of normobaric hypoxic exposure, potentially resulting in reduced blood buffer capacity and impaired anaerobic exercise performance. Additionally, our results suggest that female participants may have a greater reduction in [HCO_3_^−^] than male participants. We also assessed differences in hypoxia-induced impairment of high-intensity performance parameters depending on the duration of acute hypoxic exposure (1 h vs. 12 h normobaric hypoxic exposure) to investigate for a potential influence of [HCO_3_^−^] loss on anaerobic exercise performance. Indeed, anaerobic exercise performance output is impaired under normobaric hypoxic condition within the present investigation. However, long-term, acute exposure of 12 h to a simulated altitude of 3,000 m does not cause more pronounced impairment of anaerobic, high-intensity exercise, but results in a lower blood lactate response compared with short-term exposure of 1 h to hypoxic conditions. This finding indicates ongoing acclimatization processes, generally defined as the lactate paradox.

## Data Availability

The datasets generated and analyzed during the current study are available in the figshare repository [10.6084/m9.figshare.11663889].
